# The Landscape Of Alpha Fetoprotein In Hepatocellular Carcinoma: Where Are We?

**DOI:** 10.7150/ijbs.64537

**Published:** 2022-01-01

**Authors:** Xin Hu, Ronggao Chen, Qiang Wei, Xiao Xu

**Affiliations:** 1 Department of Hepatobiliary and Pancreatic Surgery, The Center for Integrated Oncology and Precision Medicine, Affiliated Hangzhou First People's Hospital, Zhejiang University School of Medicine, Hangzhou 310006, China.; 2 Zhejiang University Cancer Center, Hangzhou, 310058, China.; 3 Department of Hepatobiliary and Pancreatic Surgery, The First Affiliated Hospital, Zhejiang University School of Medicine, 79 Qingchun Road, Hangzhou, 310003, China.; 4 Institute of Organ Transplantation, Zhejiang University, Hangzhou, 310003, China.

**Keywords:** Alpha-fetoprotein, Hepatocellular carcinoma, Liver transplantation, Chimeric antigen receptor (CAR) T-cell therapy, T-cell receptor-engineered T-cell therapy

## Abstract

Hepatocellular carcinoma (HCC) is the most common primary liver cancer and has been acknowledged as a leading cause of death among cirrhosis patients. Difficulties in early diagnosis and heterogeneity are obstacles to effective treatment, especially for advanced HCC. Liver transplantation (LT) is considered the best therapy for HCC. Although many biomarkers are being proposed, alpha-fetoprotein (AFP), which was identified over 60 years ago, remains the most utilized. Recently, much hope has been placed in the immunogenicity of AFP to develop novel therapies, such as AFP vaccines and AFP-specific adoptive T-cell transfer (ACT). This review summarizes the performance of AFP as a biomarker for HCC diagnosis and prognosis, as well as its correlation with molecular classes. In addition, the role of AFP in LT is also described. Finally, we highlight the mechanism and application prospects of two immune therapies (AFP vaccine and ACT) for HCC. In general, our review points out the prevalence of AFP in HCC, accompanied by some controversies and novel directions for future research.

## Introduction

Hepatocellular carcinoma (HCC), which is closely related to chronic liver disease, accounts for most primary liver cancers (representing 70 to 85%)^1^. It has the sixth-highest incidence among all cancers and is the third leading cause of cancer-related death globally^2^-^4^. The 5-year survival rate of HCC is only approximately 15%^5^, ^6^. Its high mortality is considered to be a result of late detection, therapy resistance, a high recurrence rate after treatment and significant molecular heterogeneity^7^.

Progress has been made in drug chemotherapy, radiotherapy and interventional therapy due to further understanding of the etiology pathogenesis of HCC. However, treatments for patients with advanced HCC are still limited, and liver transplantation (LT) remains the best curative method for HCC^8^. For example, sorafenib, a multikinase inhibitor, demonstrated an increased survival rate accompanied by an increased incidence of adverse events^9^. The heterogeneity of HCC is an obstacle to the precise diagnosis and treatment. Given the individual differences, the achievement of early diagnosis and therapy requires specific biomarkers, an understanding of molecular subtyping, precise criteria for candidate selection for various therapies and the development of immunotherapy.

**Figure 1:**
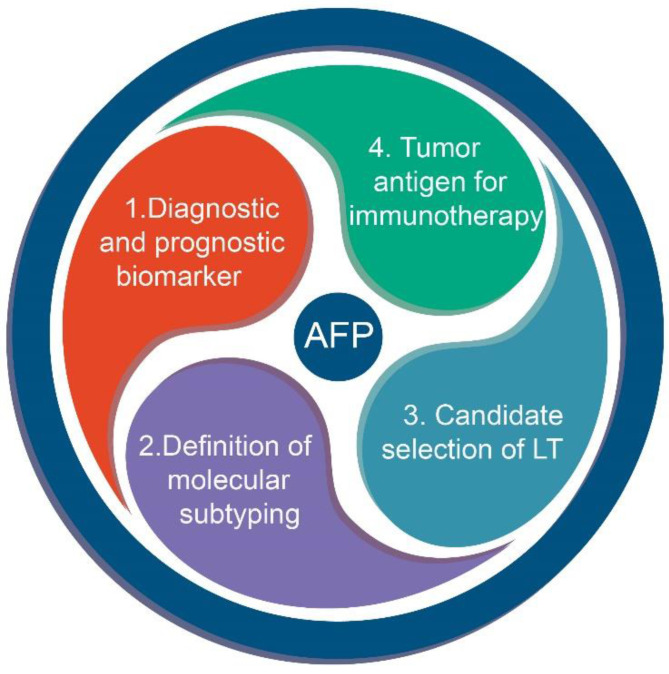
The role of AFP in HCC.

Current studies are aiming at selecting biomarkers to improve early diagnosis and prolong the survival of patients with HCC. The most common serologic marker of HCC is alpha-fetoprotein (AFP)^10^, ^11^. Identified in human fetal sera by Bergstrand and Czar in 1956, AFP acts as a transporter for several ligands, such as bilirubin, fatty acids and possibly some drugs^12^. Normally, its levels drop sharply after birth and remain at a low level thereafter. It has been used for screening, diagnosis, prognostication and therapeutic evaluation of HCC since it was identified as an oncofetal biomarker. In addition, it is also applied as an indicator in some new criteria for the selection of LT recipients, such as the Hangzhou criteria^13^. Over the past decade, some progress has been made in the use of AFP based on clinical and basic studies. In addition to being a biomarker for HCC and LT, it might be employed for immune therapy^14^ as well as for defining the HCC molecular classes^15^ (Figure 1).

## AFP is overexpressed in HCC

**Figure 2:**
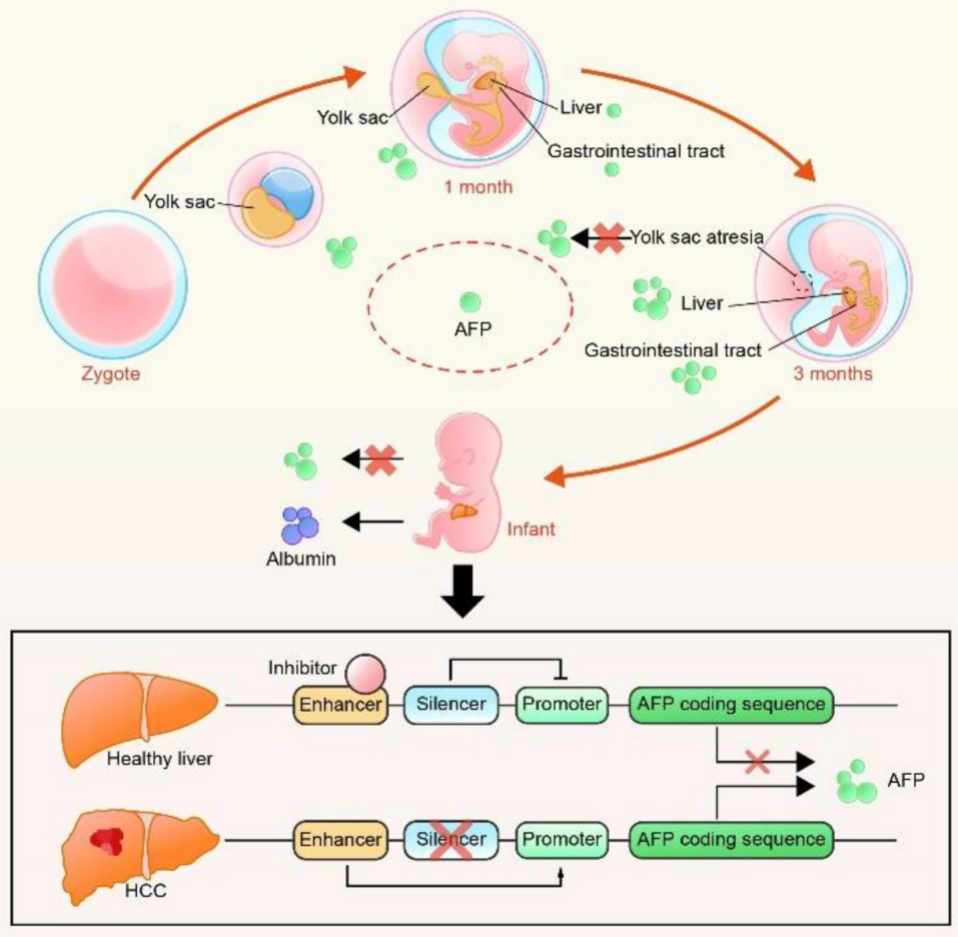
The origin of AFP in different periods and the hypothesis of AFP overexpression in HCC. AFP is produced by the yolk sac from zygote to three months of pregnancy and by fetal liver and gastrointestinal tract from the fourth week of pregnancy. After birth, AFP is gradually replaced by albumin. The re-secretion of AFP in HCC is thought to be a coaction of enhancers and silencers.

AFP is produced by the yolk sac during the first trimester of pregnancy. Then, as the sac becomes atretic, the production of AFP decreases rapidly. After the fourth week of pregnancy, the fetal liver and gastrointestinal tract begin to secrete AFP, which is sustained throughout the embryonic development period^16^. In healthy individuals, AFP is maintained at a low level throughout the lifespan but it is aberrantly expressed in HCCs. The AFP gene, which belongs to the albumin gene family, is located on the long arm of chromosome 4 of humans (4q11-q13), and it has two independent enhancer and silencer regions^17^. Several studies have indicated that a block of enhancer inhibition and deletion of the silencer leads to the restoration of promoter activity, resulting in the overexpression of AFP^8^, ^18^ (Figure 2).

## AFP is used as a biomarker in HCC

Although many scientists are now seeking new biomarkers due to the controversy regarding the utility of AFP, it remains the most universally used biomarker for HCC. It has been confirmed that persistently increased AFP level, which has been proven to be associated with an aggressive histological morphology (vascular invasion, poorly differentiated and satellitosis), are a hazardous factor for HCC^2^, ^8^, ^19^, ^20^. Current studies have discussed the critical role of AFP as a biomarker in HCC for surveillance, diagnosis and prognostication^4^, ^7^, ^8^. However, the fact that AFP could also be elevated in other benign liver diseases sparked controversy about the use of AFP for HCC surveillance^21^-^23^. Given these reasons, recent studies have tried to combine AFP with other factors. In a meta-analysis, Tzartzeva et al. compared the efficiency of surveillance imaging with or without AFP for the early detection of HCC in patients with cirrhosis and found that the former improved the sensitivity from 45% to 63%^24^. Based on the other meta-analysis, a score based on AFP, AFP-L3 and DCP was also confirmed to have a superior ability for early diagnosis^25^, ^26^. Other studies have proposed a combination of AFP with platelets and age^27^, CEA and CA-199^28^, microRNAs^29^, ^30^ and protein induced by vitamin K absence/antagonist-II (PIVKA-II)^31^. The satisfactory results of these studies have led to recommendations that AFP should be integrated with other factors by some guidelines for HCC screening^10^, ^32^, ^33^. For predicting patient outcomes, baseline AFP levels and dynamic AFP monitoring could reflect the prognosis and the response to different treatments. Three phase III studies identified high AFP as a prognostic factor of a worse overall survival (OS)^34^, ^35^. However, Giannini et al. pointed out that AFP had no prognostic significance in those with well-compensated cirrhosis and a single, small HCC (≤ 3 cm) treated with curative intent^36^. Moreover, the use of AFP was found to be valid in the therapeutic evaluation of drug treatment (lenvatinib^35^, regorafenib^37^, cabozantinib^38^ and ramucirumab^39^). For example, lenvatinib showed a superior curative effect than sorafenib when the cutoff value of AFP was 200 ng/mL (HR: 0.78, 95% CI: 0.63-0.98)^35^. There is no doubt that AFP has utility in HCC screening and prognostication, but additional studies need to be conducted to explore its appropriate usage and scope of application.

It was reported that 30% of HCC patients remained AFP-negative (<20 ng/mL)^40^, and many institutions no longer recommend the use of AFP during HCC surveillance^10^, ^41^, ^42^. Compared with AFP-positive patients, AFP-negative patients might have smaller tumor sizes, lower recurrence rates, superior liver function and a better Edmondson-Steiner grade with complete neoplasm capsules^43^, ^44^. In addition, AFP negativity was found to be a favorable predictor of LT eligibility, which means that these patients would benefit more from LT^45^. Currently, for patients with a significant increase in AFP, a liver biopsy can be performed directly to confirm the diagnosis. Therefore, most studies have focused on distinguishing AFP-negative HCC from benign liver diseases (liver cirrhosis (LC), chronic hepatitis and so on) and normal groups with no significant increase in AFP. Several potential biomarkers and laboratory tests have been identified for the diagnosis and prognostication of AFP-negative HCC (Table 1).

Stable, detectable serological biomarkers for AFP-negative HCC have been widely explored, and most of them are proteins^46^-^50^ and genes^51^-^53^. Liu et al. concluded that des-gamma-carboxyprothrombin (DCP) can distinguish AFP-negative HBV-related HCC from chronic HBV infection (AUC = 0.731) or LC (AUC = 0.685)^48^. Several studies have attempted to combine multiple biomarkers^54^-^61^ or serological examinations^62^-^66^. With the help of proteomics technology, researchers have identified some abnormally expressed proteins that were verified in various cancers^67^-^70^ to construct a logistic regression model, which had good performance in distinguishing AFP-negative HCC 60. A logistic regression model consisting of LHPP^71^-associated microRNAs (miR‐363‐5p and miR‐765) and PIVKA-II exhibited a high identification value with an AUC of 0.930^57^. The ratio of fucosylated serum paraoxonase 1 to the total serum paraoxonase 1 (Fuc-PON1)^58^ as well as the combination of fibrinogen to prealbumin ratio (FPR) and gamma-glutamyl transpeptidase to platelet ratio (GPR)^59^ were proven to have diagnostic potential (AUC = 0.78, sensitivity = 62.2%, specificity = 67.7% and AUC = 0.98, sensitivity = 91.1%, specificity = 96.5%, respectively). Moreover, Wang et al. constructed a nomogram including body mass index (BMI), oncology indicators and liver function indicators, while Huang et al. applied cirrhosis, alkaline phosphatase (ALP), tumor size, microvascular invasion, satellite lesions and tumor differentiation to build a nomogram^64^, ^65^. These models had a more accurate predictive and superior discriminative power relative to the conventional method, with C-indexes for OS prediction of 0.807 (95% CI: 0.770-0.844) and 0.742 (95% CI: 0.684‐0.800), respectively.

Recently, many studies have pointed out the lack of an accurate diagnosis when using AFP, but its isoforms were found to be a specific alternative. There are three various AFP isoforms (AFP-L1, AFP-L2, and AFP-L3) based on the binding capacity of lens culinaris agglutinin (LCA). Among them, AFP-L3, also known as lens culinaris-reactive AFP, is the main isoform in HCC patients, especially in small HCCs (< 3 cm)^72^. AFP-L3 was identified to be related to poorly differentiated and advanced HCC^73^. It can be detected in early-stage HCC, especially when it is supplied by the hepatic artery, and AFP-L3-positive HCC is more likely to have an early metastasis and rapid growth^74^. Currently, many studies have applied AFP L3 as an adjuvant marker to improve the accuracy and completeness of early diagnosis of HCC^26^, ^75^, ^76^.

To date, most of these results were acquired from retrospective, single-center studies with small samples, and there is a lack of prospective, large-sample and multicenter studies to confirm their value.

## AFP is associated with HCC molecular classes

As a heterogeneous disease, patients diagnosed with HCC have diverse clinical features and disease progression levels^15^, ^77^. With the continuous development of bioinformatics, especially the progress in gene sequencing technology, the classification of HCC is no longer limited to the histopathological level. Several new molecular classifications defined by AFP combined with other indicators have been successively discovered and validated. These distinct classifications are associated with different morphological phenotypes and clinical characteristics, which are linked to specific genetic mutations and signaling pathways^78^, ^79^.

### AFP is used to define novel classes

The expression of epithelial cell adhesion molecule (EpCAM) is positive in the majority of hepatocytes in the embryonic liver. However, in adults, it is negative in hepatocytes and positive in the bile duct epithelium^80^. EpCAM+ HCC exhibits hepatic cancer stem cell-like, highly invasive and tumorigenic features^81^, ^82^. Yamashita et al. classified HCC into four subtypes by EpCAM and AFP (EpCAM^-^ AFP^-^, EpCAM^-^ AFP^+^, EpCAM^+^ AFP^-^ and EpCAM^+^ AFP^+^ HCC) with the name of mature hepatocyte-like HCC, hepatocytic progenitor-like HCC, bile duct epithelium-like HCC and hepatic stem cell-like HCC^83^, ^84^. Apparent differences existed in the transcriptome of these subtypes, and AFP^+^ HCC (EpCAM^-^ AFP^+^ and EpCAM^+^ AFP^+^ HCC) was more likely to have a poor prognosis, advanced TNM stages and vascular invasion^84^. The S2 subclass identified by Hoshida et al. showed that increased AFP levels were also distinctly enriched in a signature of EpCAM positivity^85^. Recently, some scientists have further explored the molecular mechanisms and potential therapeutic targets of EpCAM^+^ AFP^+^ HCC. Wei et al. discovered that MAGE-A9 (a specific cancer testis antigen), whose anomalous expression was correlated with enhanced tumor proliferation and metastases, was increased in EpCAM^+^ AFP^+^ HCC characterized by hepatic stem/progenitor cells, indicating that MAGE-A9 might perform a role in regulating stem cell-like feature and act as an underlying therapeutic target^79^. Furthermore, Takai et al. conducted a genome-wide RNAi screen to explore genes with a synthetic lethal interaction with EpCAM and filtered out PMPCB, which encodes proteins to maintain the function of mitochondria as a potential target^82^. Moreover, based on the expression of AFP and CD133 (a typical stem cell marker), Dai et al. classified HCC into four groups (CD133^+^AFP^+^, CD133^-^AFP^-^, CD133^+^AFP^-^ and CD133^-^AFP^+^ HCC) with significantly distinct clinicopathological features and prognosis^78^.

### AFP is abnormally expressed in several classes

Apart from defining the novel classes, AFP was also proven to be increased or decreased in several molecular classifications (Table 2).

#### HCC mutated with CTNNB1

CTNNB1 involved in the Wnt/β-catenin signaling pathway is a prevalent mutation gene in HCC^86^-^89^. Calderaro et al. indicated that CTNNB1 mutations defined a specific cholestatic, low inflammatory infiltrate levels and a well-differentiated subtype of HCC with a lower expression of AFP compared with the nonmutation group^15^. Another study found that an HCC subtype overexpressing AFP (median serum level, 472 ng/mL) exhibited tyrosine kinase activation (IGF1R, RPS6 and Akt phosphorylation), decreased frequencies of CTNNB1 exon 3 mutation and 6q loss, increased frequencies of 4q and 13q loss and significant macrovascular invasion^90^.

#### HCC mutated with TP53 and a novel subtype (MTM-HCC)

As a hallmark in DNA repair, genomic stability and apoptosis regulation, TP53 mutation was found to be correlated with AFP positivity, as there were 50.00% (12/24) of AFP-positive HCC in the TP53 mutation group and 20.69% (6/29) in the wild type (p < 0.05)^91^. Another study identified a prognostic protein biomarker, ADH1A (oxidoreductase activity), associated with metabolic reprogramming, and HCC with high ADH1A showed reduced TP53 mutations and lower AFP levels^92^. Similarly, Yang et al. pointed out that the low-AFP subclass C1 had numerous enriched metabolism-associated biological processes (especially the urea cycle), a significantly lower mutation frequency of TP53, and notable cabozantinib resistance^93^. In addition, TP53 mutation was proven to be associated with a novel histological subtype called "macrotrabecular-massive HCC (MTM-HCC)", which was designated by Calderaro et al. and characterized by a predominant macrotrabecular architecture involving more than 50% of the tumor, high AFP serum levels (AFP > 100 ng/ml, P < 0.02) and poor recurrence-free survival^15^, ^94^. Logistic and multivariable cox regression analyses were performed and found that a high serum AFP levels was an independent feature and predictor (OR: 4.4, 95% [CI]: 1.3, 16; P = 0.02) of the MTM-HCC subtype^95^, ^96^.

#### Other classes

Six robust subgroups of HCC (G1-G6) were identified by Boyault et al. after investigating 57 HCCs by global transcriptome analysis, and HCCs involved in G1-G3, which are known to be characterized by chromosomal instability and high cell proliferation, were correlated with elevated AFP levels (AFP > 100 ng/mL; P < 0.001)^15^, ^97^. Glypican-3 (GPC3), a protein that can stimulate the proliferation and migration of tumor cells through the activation of Wnt signaling in HCC^98^, was applied by Xue et al. to divide 316 patients into GPC3+ and GPC3- phenotypes^99^. The results revealed that there was a significant difference in serum AFP levels between the two groups^99^.

Elevated levels of AFP indicate aggressive tumor pathologic characteristics and a poor prognosis^85^, ^100^, ^101^. Currently, some genes recognized as signatures in novel molecular classifications have been identified. Combined with these genes or their coding proteins, the alteration of AFP levels might show better performance in defining new subtypes.

## Applying AFP for candidate selection and predicting the recurrence of LT

Currently, LT remains the best treatment for HCC because it eliminates carcinogenic background. Whether patients obtain effective disease mitigation after LT relies on the use of accurate criteria for candidate selection. At present, the Milan criteria (MC) (single tumor nodule, tumor diameter < 5 cm or no more than three tumor nodules, none exceeding 3 cm in diameter) is the most widely used in 95% of countries to select suitable candidates^102^, ^103^. However, several studies confirmed that patients beyond the MC had comparable post-LT survival rates, suggesting that MC might preclude access to LT for those who might benefit^13^, ^104^, ^105^. In addition, researchers have found a powerful predictive ability of some biomarkers for LT outcomes, especially AFP. Distinct evidence has shown that the post-LT survival rate declines with increasing AFP levels^103^, ^106^, ^107^. Hence, scientists have employed AFP in candidate selection and prognostication to relax the criteria and expand the donor pool (Table 3).

### AFP with tumor morphology

AFP is most commonly used in combination with tumor morphology. A Korean group created a revised scoring system based on tumor size, tumor number and pretransplant AFP levels (< or =20, 20.1 to 200, 200.1 to 1000, >1000 ng/mL), allowing an expansion for candidate selection without adverse outcomes^108^. Similarly, another two criteria, named the "Model of Recurrence After Liver Transplantation" (MORAL) and New York/California (NYCA) scores developed by Halazun et al., provided highly accurate tools for candidate selection and forecasting recurrence^109^, ^110^.

Several studies have focused on total tumor diameter (TTD) or total tumor volume (TTV) rather than single tumor features. Zheng et al. designed the Hangzhou criteria, which included AFP, TTD and histopathologic grade, for candidate selection, indicating the possibility of LT for those who were beyond MC but fulfilled the Hangzhou criteria and pointing out that AFP >100 ng/mL was an independent prognostic factor among them^13^. In some cases, the Hangzhou criteria was also considered as a downstaging criteria for HCC patients before LT to lower the threshold for LT^111^. Then, a team from Italy proposed a score containing AFP and TTD (the AFP-TTD score) but no histopathologic features, which simplified the Hangzhou criteria^112^. With no need for a tumor biopsy, it could avoid bleeding, tumor seeding and unnecessary surgery. Besides, by utilizing the same three characteristics, Duvoux et al. proposed an AFP model whose cutoff values were 100 ng/ml and 1000 ng/ml^113^. Its superiority of strong predictability has been validated in different populations^114^-^116^. Notably, this model was innovatively used to predict the recurrence rate in patients with viral hepatitis-related cirrhosis who had received LT for HCC^107^. Other scores combining AFP with TTD or TTV were also proposed^117^-^119^. Interestingly, Mazzaferro et al. applied the sum of the number and size of tumors (in centimeters) to replace TTV/TTD to build a Metroticket 2.0 Model, expanding the idea of tumor morphology^120^.

### AFP with the model for end-stage liver disease (MELD)

The end-stage liver disease (MELD) model is used for evaluating liver function reserve and prognosis in patients with chronic liver disease. Integrating MELD into the evaluation system allowed for a complete assessment of patients' preoperative status since many patients had a background of cirrhosis. In this situation, some models including MELD were designed^120^-^125^. Among them, Vitale et al. established a model using transplant benefit as the common endpoint to re-establish allocation equity in patients with and without HCC^123^. They created a "MELD equivalent" that matches HCC patients to non-HCC patients by the same numerical MELD score and developed the equation: HCC-MELD (1.27∗MELD - 0.51∗logAFP+4.59), whereby the same transplant benefit between the two groups was achieved.

### AFP with modified Response Evaluation Criteria in Solid Tumors (mRECIST)

Attention to locoregional therapy (LRT) has increased because effective preoperative LRT predicts a low recurrence rate. Complete response (CR), partial response (PR), stable disease (SD), and progressive disease (PD) (MRECIST) is widely applied to measure the response to LRT^126^, ^127^. The Time-Radiological-response-Alpha-fetoprotein-Inflammation (TRAIN) score regards LRT as one of the risk variables in its formula^128^. Another study pointed out that the Metroticket 2.0 criteria^120^ affiliated with mRECIST enhanced its prediction ability^129^.Moreover, Lai et al. made use of pre-LT LRT to stratify the survival rate of LT and to improve the equity of liver allocation^124^, ^130^. In general, patients with a good response to LTR are likely to gain better post-LT prognostics, suggesting that LRT is a valuable factor for LT decisions.

### AFP with other factors

Some novel indicators have been investigated for integration with AFP, such as 18F-fluorodeoxyglucose positron emission tomography (18F-FDG PET)^131^, ^132^ and plasma metabolomics profiling^133^. In addition, several criteria established and validated previously were combined with AFP for the purpose of minimizing the risk of post-LT tumor recurrence^134^, ^135^. Grąt et al. cited University of California, San Francisco (UCSF) criteria, Up-to-7 criteria and AFP levels <100 ng/ml to build a score^134^. It exhibited the superior predictive power since patients fulfilling two criteria with AFP levels <100 ng/ml showed an excellent 5-year recurrence-free survival (100.0%). This score, named the Warsaw proposal, was verified in a total of 240 HCC patients^136^.

### The value of dynamic AFP

The variation in AFP from pre-LT to post-LT might be better to evaluate disease progression. Post-LT AFP levels not decreasing to 20 ng/ml were proved to be a risk factor for recurrence by Xu et al^137^. Lai et al. conducted a retrospective study on 422 HCC patients who underwent LT, confirming that an AFP slope > 15 ng/mL/month was a unique independent predictive factor for HCC outcome^130^. A similar result was also found in another study^128^. Later, an AFP slope >7.5 was shown to be significantly related to HCC recurrence (HR, 3.0; P=0.03) and was also associated with microvascular invasion (OR, 6.8; P=0.008)^138^.

**Figure 3:**
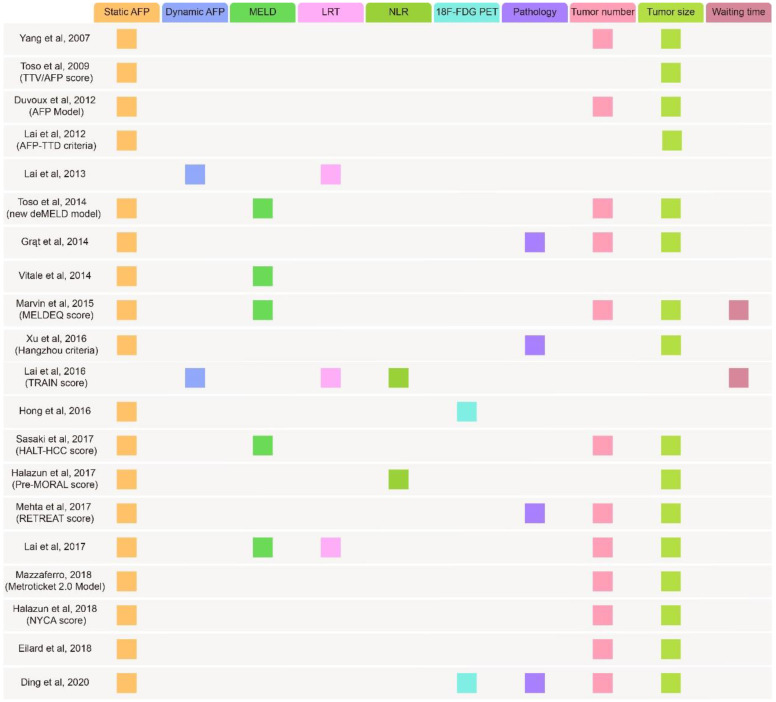
Summary of several metrics used in proposed criteria. TTV: total tumor volume; TTD: total tumor diameter; deMELD: dropout equivalent calculated equivalent Model for End-Stage Liver Disease; MELDEQ: equivalent Model for End-Stage Liver Disease; TRAIN: time-radiological-response-alpha-fetoprotein-inflammation; HALT-HCC: Hazard Associated with Liver Transplantation for Hepatocellular Carcinoma; MORAL: model of recurrence after liver transplant; RETREAT: risk estimation of tumor recurrence after transplant; NYCA: New York/California; MELD: model for end‐stage liver disease; LRT: loco-regional treatment; NLR: neutrophil-to-lymphocyte ratio; ^18^F-FDG PET: ^18^F-fluorodeoxyglucose positron emission tomography.

Since multiple studies have confirmed the predictive value of the AFP level and offered several reliable criteria (Figure 3) containing it^139^, ^140^, fairness of liver allocation and prediction of the outcome of LT have been significantly improved.

## The role of AFP in immune therapy

### AFP serves as the biomarker for checkpoint inhibitor

In the process of tumor occurrence and development, immune checkpoint has become one of the main reasons for immune tolerance. Immune-checkpoint inhibitor (ICI) promotes the host to recognize tumor antigen and to generate an immune response by ceasing the co-inhibitory signaling^141^. The arrival of ICI as a new milestone for HCC treatment has led to a conceptual transform of therapeutic strategy. Recently, several studies have proved that the change of AFP could accurately reflect the therapeutic effect of ICI. Spahn et al. conducted a study contained 67 patients received nivolumab and 32 patients received pembrolizumab to explore the biomarkers to predict response to ICI^142^. They pointed that the patients whose AFP < 400 µg/L at the beginning of ICI treatment were more likely to have complete response. Besides, AFP < 400 µg/L was related to a longer median progress-free and overall survival. Similarly, Post-treatment decline in serum AFP levels were also proved to be a predictor of prognosis^143^-^145^.

### AFP performs as a tumor antigen for immune therapy

Increasing evidence has shown that infiltrating immune cells in HCC tissue, which form the tumor immune microenvironment, play an important role in tumor proliferation and metastasis^146^-^148^. HCCs belonging to different immune-specific classifications and immune cell infiltrations might refer to distinct outcomes of therapies. Kurebayashi et al. identified that patients in a cytokeratin 19^+^-associated immune-high subtype had a better prognosis^147^. Hence, developing various immunotherapies aiming at different HCC classifications or using biomarkers to select appropriate patients is particularly important.

Immunotherapy efficiency depends on the recognition of tumor-specific antigens by the autoimmune system. The re-expression of AFP is observed in approximately 70%-80% of HCC patients but is not observed in healthy individuals after birth^14^, ^149^. In addition, AFP has been proven to promote tumor proliferation through the initiation of the cyclic AMP-protein kinase A pathway, Ca2+ influx and apoptotic signal transduction mediated by caspase‐3^150^-^152^. AFP can also mediate HCC immune escape by altering the proportion of CD4+ T/CD8+ T cells^153^ and inhibiting dendritic cells (DCs)^154^ and natural killer (NK) cells^155^. These features make AFP itself an appropriate therapeutic target. However, immune tolerance results in a low immune response to AFP despite the immune system being exposed to high plasma levels of AFP^150^, ^156^. The crucial point of mounting effective antitumor immunity is to ameliorate the low affinity of the immune system to AFP. Many approaches containing recombinant plasmid DNA, adoptive transfer of tumor-specific T cells and chimeric virus-like particles have been proposed to improve the immune response^14^, ^157^-^159^.

#### AFP-based cancer vaccine

HCC vaccines are designed to target tumor-specific antigens to induce an effective immune response, aiming to prevent tumor proliferation and even eliminate it. AFP is considered a favorable target due to its immunogenicity and specificity. The AFP vaccine presents the AFP epitope polypeptides to antigen presenting cells (APCs), generating multiple AFP-specific cytotoxic T lymphocytes (CTLs) to induce tumor immunity. At present, a variety of AFP vaccines have been created, such as DC vaccines^160^-^163^, DNA vaccines^164^ and peptide vaccines^165^-^167^, which have been continuously applied to HCC mouse models and clinical trials.

DC vaccines exhibit favorable application prospects due to their specificity and effectiveness for immunotherapy of HCC. Vollmer et al. first reported genetically engineered and AFP-transduced DCs that were injected into C57BL/6 mice and elicited effective T-cell immune responses^160^. More recently, scientists have attempted to boost the antigen-presenting function of DCs. Methods such as zoledronic acid stimulation^168^, DC-derived exosomes (DEXs)^161^, ^162^ and coculture with IL-2 and GM-CSF^169^ could promote the secretion of valid interferons (IL9, IL15 and TNF) to enhance tumor immunity.

It has been proven that exosomes are involved in the biological behavior. DEXs were then discovered to express major histocompatibility complex class I and II (MHC I and II) and costimulatory molecules^170^, ^171^. Therefore, Lu et al. monitored the tumor growth and immune microenvironment of three HCC mouse models after using exosomes derived from AFP-expressing DCs (DEX_AFP_)^161^. It induced more powerful antigen-specific immune responses, which were demonstrated by the prevention of tumor proliferation, a prolonged survival time and an ameliorative tumor microenvironment (increased levels of IFN-γ, IL-2 and CD8^+^ T lymphocytes). Later, the same conclusions were found when Li et al. stimulated naive T cells with DEXs generated by peripheral blood-derived DCs loaded with the recombinant adeno-associated viral vector (rAAV) -carrying AFP gene^162^. In addition, two researchers applied tumor antigen-pulsed dendritic cells as an immunotherapy to treat HCC patients and obtained encouraging therapeutic effects^163^, ^172^. However, it is worth noting that DEXs may transfer their immunogenicity to other APCs due to secretion and uptake of exosomes, leading to antigen cross-presentation among APCs. In brief, DEXs are the novel idea for a cell-free vaccine, and their combination with DCs might be feasible.

Polypeptide vaccines can be synthesized *in vitro* without the involvement of viral vectors, rendering them safe and easy to produce. Compared to oligopeptides, the higher relative molecular weight and the stronger immunogenicity of polypeptides could make CTLs more efficacious [192]. Tam et al. described a multiple antigen peptide (MAP) system to synthesize a peptide-antigen matrix by a solid-phase method^173^. Recently, two phase I clinical studies have been conducted to investigate the safety and efficacy of AFP peptide vaccination for patients with advanced HCC^166^, ^167^. Nakagawa et al. injected AFP-derived peptides (AFP_357_ and AFP_403_) into 15 patients and found that one patient had a complete remission, eight patients had tumor suppression, and none had adverse events^166^. Another study employed a combination of peptide vaccination and radiotherapy, showing a 33% response rate and 66% disease control rate with no side effects^167^. To boost T-cell responses, Li et al. made use of heat shock protein 72 (HSP72) and AFP epitope peptide (AFP-P) to construct a peptide vaccine and then immunized BALB/C mice^174^. Compared to those immunized with AFP-P or HSP72 alone, mice immunized with HSP72/AFP-P developed more IFN-γ-producing CD8+ T cells and their tumor volume was smaller. Similar results were also found when crosslinking the AFP epitope peptide with heat shock protein 70 functional peptide or glycoprotein 96^165^, ^175^, ^176^.

#### AFP as a target for Chimeric antigen receptor (CAR) T-cell and T cell receptor (TCR) T-cell therapy

**Figure 4:**
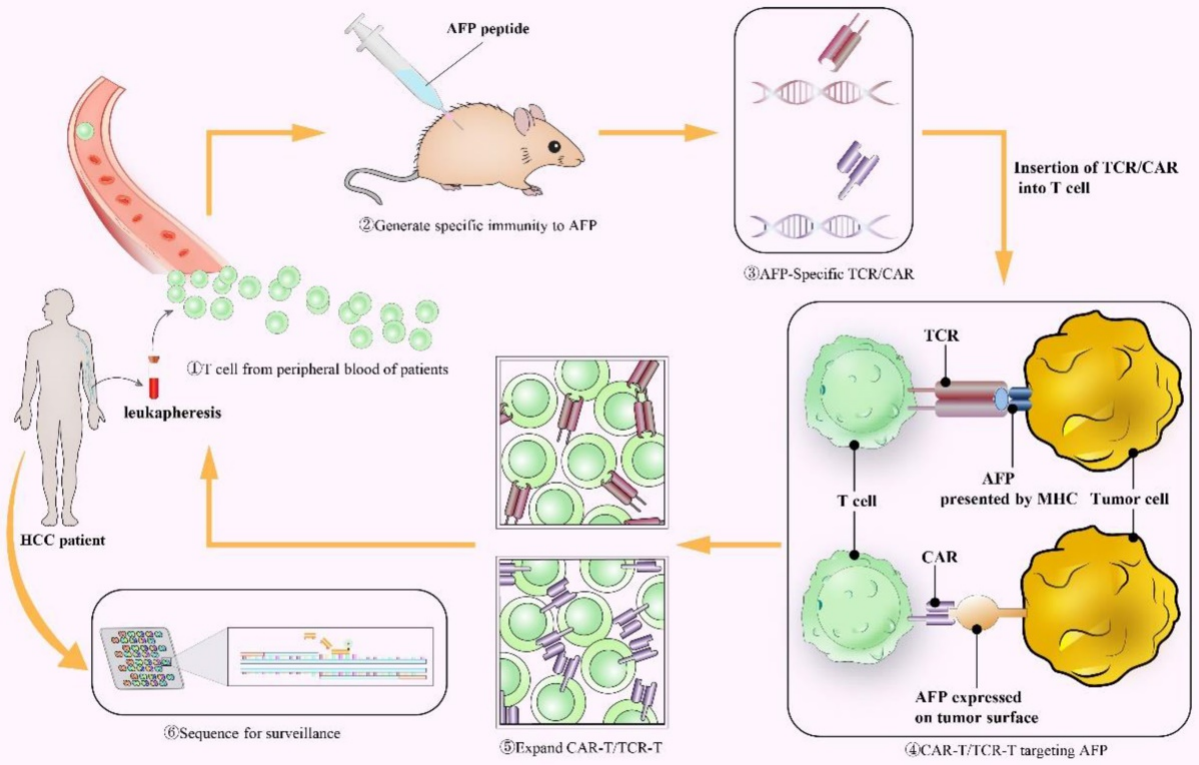
The process of AFP performing as a tumor antigen in CAR T-cell or TCR T-cell therapy. CAR: Chimeric antigen receptor; TCR: T cell receptor; MHC: major histocompatibility complex.

CAR T-cell therapy, which grafts genetically engineered receptors onto host T cells to target tumor-associated antigen (TAA), represents a remarkable advance in immunotherapy for cancer (Figure 4). It made modified T cells MHC-unrestricted. The FDA has approved two CAR-T therapies targeting CD19 antigen (Kymriah and Yescarta) for the treatment of acute lymphoblastic leukemia (ALL) and diffuse large B-cell lymphoma (DLBCL) due to their powerful antitumor effects^177^, ^178^. Liu et al. generated a novel CAR (ET1402L1) that specifically bonded to the AFP_158-166_ peptide presented by HLA-A*02:01^179^. T cells could suppress HLA-A*02:01^+^/AFP^+^ tumor growth *in vivo* and *in vitro* after being transduced by this AFP-CAR. This result also suggested that local injection of AFP-CAR T cells promoted a more intense and sustained immune response^179^ so that local treatment may be a better method. The AFP-CAR could bind to the peptide-MHC complex, intracellular antigens and secreted protein products that could not be recognized by traditional CAR.

Some ideal TAAs, which are expressed on all tumor cells but hardly express on normal tissues, are found inside the cell and must be presented to the cell surface by the MHC to activate the immune response^180^, ^181^. The TCR utilizes heterodimers to recognize intracellular or cell surface MHC-restricted TAAs, while traditional CAR cannot (Figure 4). Thus, the first and most critical step of TCR T-cell therapy is to engineer a TCR that specifically binds to the AFP peptide-MHC complex. Recently, several studies have identified optimized TCRs that can recognize AFP/HLA-A*02^+^ tumor cells^182^-^184^. Zhu et al. immunized HLA-A2 transgenic AAD mice with the AFP_158_ epitope peptide to generate AFP_158_-specific CD8^+^ T cells with TCR diversity and transduced three pairs of TCR genes into human T cells^182^. The results showed that both mouse CD8^+^ T cells and engineered human T cells could kill HLA-A2^+^ AFP^+^ HepG2 tumor cells without targeting normal primary hepatocytes *in vitro*. Then, Luo et al. excluded two of the above three TCRs^182^ due to their underlying cross-reactivity, and the remaining TCR with optimal affinity, efficiency and safety was applied to an early clinical trial (NCT03971747)^185^. Similar findings (an increased number of IFN-γ secretion T cells and cytotoxicity toward tumor cells) were achieved when Sun et al. infected nonspecific T cells with a lentiviral vector constructed by cloned TCR genes of AFP-specific CTLs^183^. Furthermore, on the basis of AFP-specific TCRs, Docta et al. employed a combination of physicochemical and cell biology methods to adjust the TCR affinity^184^. These TCRs were validated among normal and malignant cells in different tissues, cell types and HLA alleles. Instead of HLA-A * 02: 01, HLA-A * 24: 02 was found to be more common in Asian populations, so Li et al. distinguished the HLA-A*24: 02-restricted peptide KWVESIFLIF (AFP_2-11_) to create a specific TCR (KWV3.1)^186^.

CAR T-cell therapy has high specificity and effectiveness, as it is not MHC restricted, but it cannot recognize intracellular antigens, while TCR T-cell therapy has a wider range of targets but is limited by MHC molecules. Studies have proven their remarkable antitumor effect. However, multiple TAAs, including AFP, were not 100% tumor-specific, and promiscuous recognition of unassociated epitopes of normal proteins might cause off-target reactivity of both therapies, which could cause serious systemic toxicity. Hence, Cai et al. measured the off-target cross-reactivity of three AFP-specific TCRs^187^. Several other peptides (ENPP1_436_ and RCL1_215_) were able to cross-activate these TCRs, but they required higher concentrations (approximately 250 times and 10,000 times, respectively) than AFP to fulfill the same level of response. Making CAR or TCR recognize multiantigen complexes simultaneously^188^ or inserting suicide genes that could be activated when off-target reactivity occurs^189^ is considered a remedial action to overcome side effects. Additional studies are needed to create TCR/CAR with an ideal affinity to target high densities of AFP on HCC while not targeting low expression on nonmalignant cells.

## Conclusion and future prospective

The heterogeneity of HCC caused by multiple pathogenic mechanisms and various risk factors gives rise to limitations in diagnosis and treatment. Identified more than 60 years ago, AFP has become one of the most frequently used biomarkers in HCC and is a critical element to select patients who are suitable for LT. Several researchers have also identified its function in HCC classes. In addition, some studies have paid attention to the role of AFP as a tumor antigen to treat HCC due to its immunogenicity and universality.

Although AFP is widely used in the diagnosis and treatment of HCC, improvements are required in many fields. Does HCC molecular classification defined by AFP have therapeutic benefits? Which combination with AFP can improve its performance in LT candidate selection? What is the optimal cut value of AFP in HCC diagnosis and prognosis? Currently, quite a few expectations have been placed regarding AFP as an antigen, and some AFP vaccines, CAR-T and TCR-T are being verified in clinical trials. However, a low immune response to AFP caused by immune tolerance and off-target reactivity in CAR-T and TCR-T become obstacles. The development of original engineered AFP peptides and an understanding of the mechanisms regulating immune escape might offer a superior therapeutic effect. Besides, how to increase the affinity of AFP epitopes for CAR and TCR should be considered.

Current applications of AFP in HCC have been widely accepted, and future challenges lie in confirming its effectiveness in clinical trials. With rapid progress in research in the future, the use of AFP will be more accurate and widespread.

## Figures and Tables

**Table 1:** Biomarkers and methods of ANAC for early diagnosis.

Content	Year	Type	AUC	Sensitivity/Specificity	Population	Ref.
Cmi	2015	microRNAs	0.83	-	Asian	52
AFP-L3	2015	protein	0.61	50.0%/97.5%	Asian	56
GP73	2015	protein	0.78	66.0%/96.2%	Asian	56
Midkine	2016	protein	0.70	70.9%/62.2%	Asian/Africa	47
FAHB-M	2016	regression model	0.88	80.3%/82.9%	Asian	63
Fuc-PON1	2017	protein	0.78	62.2%/67.7%	Asian	58
TEMs	2017	monocytes	0.69	80.0%/65.5%	Asian	49
NPM1 + 14-3-3zeta + MDM2	2017	autoantibody	-	30.4%/91.6%	Asian	55
metabolomic profiles	2019	SCMs	>0.80	-	Asian	61
hematological parameters	2019	regression model	0.92	83.0%/93.1%	Asian	66
PA + D-Dimer + Fibrinogen	2020	protein	0.94	93.4%/80.8%	Asian	54
miR-363-5p + miR-765 + PIVKA-II	2020	regression model	0.93	79.4%/95.4%	Asian	57
FPR + GPR	2020	protein, platelet	0.98	91.1%/96.5%	Asian	59
PT/Fbg system	2020	clinical examination	0.68	-	Asian	62
DCP	2020	protein	0.73	50.6%/91.7%	Asian	48
P53 +MSH2 + Tm-4 + inflammatory factors + life-history traits	2020	regression model	0.91	85.2%/88.3%	Asian	60

ANHC: AFP-negative hepatic carcinoma; Cmi: miRNA classifier; GP73: golgi protein 73; FAHB-M: fluorescence intensity, alpha-fetoprotein, hepatic function test results and blood cell analyses with the model; Fuc-PON1: the ratio of fucosylated serum paraoxonase 1 to the total serum serum paraoxonase 1; TEMs: Tie2-expressing monocytes; SCMs: significantly changed metabolites; PA: pre-albumin; PIVKA-II: vitamin K deficiency or antagonist-II; FPR: fibrinogen to prealbumin ratio; GPR: gamma-glutamyl transpeptidase to platelet ratio; PT: plasma prothrombin time; Fbg: fibrinogen; DCP: des-gamma-carboxyprothrombin; MSH2: MutS homologs 2; Tm-4: tropomyosin-4.

**Table 2:** The change of AFP levels in several classes.

Subtype	AFP level	Relevant Characteristic	Signal pathway	Population	Ref.
CTNNB1 mutation	low	large size, well-differentiated, intact tumor capsule, microtrabecular and pseudoglandular chistological patterns, tumor cholestasis, a lack of inflammatory infiltrates	IL6/JAK/STAT, Wnt/β*-*catenin	European/ North American	15, 90, 97
TP53 mutation (MTM-HCC)	high	poor differentiation, macrovascular and microvascular invasion, compact histological pattern, foci of sarcomatous changes, pleomorphic and multinucleated cells, a lack of tumor cholestasis	PI3K/AKT	European/Asian	15, 91-97
G1/G2/G3 subclasses	high	high cell proliferation, chromosomal instability, female gender, hemochromatosis, HBV infection	Cell cycle, proliferation, DNA metabolism	European	97
S2	high	large size, poor-differentiated, high proliferation	MYC and AKT	Asian	85, 100
GPC3+	high	thick trabecular pattern and compact variants, vascular invasion, distant metastasis, short survival time	-	Asian	99

MTM-HCC: macrotrabecular-massive subtype of HCC; OS: overall survival; GPC3: Glypican-3.

**Table 3:** The role of AFP in LT.

Study	No.	AFP cut value	Type	Population	Ref.
Yang et al, 2007	63	≤ 20, 20.1 to 200, 200.1 to 1000, > 1000 ng/mL	candidate selection	Asian	108
Toso et al, 2009 (TTV/AFP score)	6478	400 ng/mL	candidate selection	North American	117
Duvoux et al, 2012 (AFP Model)	537	log_10_AFP (Simplified: AFP ≤ 100, 100 to 100, > 1000 ng/mL)	candidate selection	European	113
Lai et al, 2012 (AFP-TTD criteria)	158	400 ng/mL	candidate selection	European	112
Lai et al, 2013	422	AFP slope: 15 ng/mL/month	Prediction	European	130
Toso et al, 2014 (new deMELD model)	49026	400 ng/mL	candidate selection	North American	122
Grąt et al, 2014 (combination of UCSF and Up-to-7 criteria)	121	100 ng/ml; 200mg/ml	candidate selection	European	134
Vitale et al, 2014	4399	100, 100 to 100, > 1000 ng/mL	candidate selection	European	123
Marvin et al, 2015	41801	Log AFP: 0 to 1.61, 1.61 to 2.48, 2.48 to 3.93, 3.93 to 10.9 (MELD_CALC-EQ_ = 1.143MELD + 1.324 (log AFP) + 1.438 (TumorNum) + 1.194(MaxTumorSize) + c(t), where c(t) = -2/0.146 if t < 6 months and c(t) = -1/0.146 if t ≥ 6 months)	candidate selection	North American	121
Xu et al, 2016 (Hangzhou criteria)	6012	400 ng/mL	candidate selection	Asian	13
Lai et al, 2016 (TRAIN score)	179	AFP slope: 15 ng/mL/month	prediction	European	128
Hong et al, 2016	123	200 ng/ml	prediction	Asian	131
Sasaki et al, 2017 (HALT-HCC score)	420	HALT-HCC = (1·27 × TBS) + (1·85 × lnAFP) + (0·26 × MELD-Na)	prediction	North American	125
Halazun et al, 2017 (Pre-MORAL score)	339	200 ng/ml	prediction	North American	109
Mehta et al, 2017 (RETREAT score)	721	0-20, 21-99, 100-999, ≥1000 ng/ml	prediction	North American	118
Lai et al, 2017	2103	20 ng/ml, 1000 ng/ml	candidate selection	European	124
Mazzaferro, 2018 (Metroticket 2.0 Model)	1018	<200, 200-400 ng/mL, 400-1000, >1000 ng/ml	candidate selection	European	120
Halazun et al, 2018 (NYCA score)	1450	<200, 200-1000, >1000 ng/ml	candidate selection	North American	110
Eilard et al, 2018	336	<99, 100-999, >1000 ng/ml	candidate selection	European	135
Ding et al, 2020	93	144ng / ml	prediction	Asian	132

LT: liver transplantation; AFP: alpha‐fetoprotein; TTV: total tumor volume; TTD: total tumor diameter; MELD: model for end‐stage liver disease; deMELD: dropout equivalent calculated equivalent Model for End-Stage Liver Disease; UCSF: University of California: San Francisco; MELD_CALC-EQ_: calculated equivalent Model for End-Stage Liver Disease; TRAIN: time-radiological-response-alpha-fetoprotein-inflammation; LRT: loco-regional treatment; NLR: neutrophil-to-lymphocyte ratio; ^18^F-FDG PET/CT: ^18^F-fluorodeoxyglucose positron emission tomography/computed tomography; HALT-HCC: Hazard Associated with Liver Transplantation for Hepatocellular Carcinoma; TBS: tumor burden score; MELD-Na: MELD-sodium; MORAL: model of recurrence after liver transplant; RETREAT: risk estimation of tumor recurrence after transplant; NYCA: New York/California.

## Abbreviations

HCC: Hepatocellular carcinoma
LT: liver transplantation
AFP: alpha-fetoprotein
PIVKA-II: protein induced by vitamin K absence/antagonist-II
OS: overall survival
DCP: des-gamma-carboxyprothrombin
Fuc-PON1: fucosylated serum paraoxonase 1 to the total serum paraoxonase 1
FPR: fibrinogen to prealbumin ratio
GPR: gamma-glutamyl transpeptidase to platelet ratio
BMI: body mass index
LDH: lactate dehydrogenase
GGT: gamma-glutamyl transpeptidase
ALB: albumin
ALP: alkaline phosphatase
LCA: lens culinaris agglutinin
EpCAM: epithelial cell adhesion molecule
TERT: telomerase reverse transcriptase
MTM-HCC: macrotrabecular-massive HCC
GPC-3: Glypican-3
MC: Milan criteria
MORAL: Model of Recurrence After Liver Transplantation
NYCA: New York/California
TTD: total tumor diameter
TTV: total tumor volume
MELD: model of end-stage liver disease
LRT: locoregional therapy
MRECIST: complete response, partial response, stable disease and progressive disease
TRAIN: Time-Radiological-response-Alpha-fetoprotein-Inflammation
WT: waiting time
18F-FDG PET: 18F-fluorodeoxyglucose positron emission tomography
UCSF: University of California, San Francisco
DCs: dendritic cells
NK cells: natural killer cells
APCs: antigen presenting cells
CTLs: cytotoxic T lymphocytes
DEXs: DC-derived exosomes
MHC: major histocompatibility complex
DEX_AFP_: AFP-expressing DCs
rAAV: recombinant adeno-associated viral vector
MAP: multiple antigen peptide
HSP72: heat shock protein 72
AFP-P: AFP epitope peptide
CAR: Chimeric antigen receptor
TCR: T cell receptor
TAA: tumor-associated antigen
ALL: acute lymphoblastic leukemia
DLBCL: diffuse large B-cell lymphoma
